# Mechanical Evaluation of Salvage Fixation Techniques for a Lateralized Fibular Tunnel in Posterolateral Corner Reconstruction of the Knee

**DOI:** 10.1177/23259671221131303

**Published:** 2022-11-30

**Authors:** Arya Amirhekmat, Wendy E. Brown, Kyriacos A. Athanasiou, Dean Wang

**Affiliations:** * Department of Orthopaedic Surgery, UCI Health, Orange, California, USA.; † Department of Biomedical Engineering, University of California, Irvine, Irvine, California, USA.; *Investigation performed at the University of California, Irvine, Irvine, California, USA*

**Keywords:** posterolateral corner, fibular head, ligament reconstruction, staple

## Abstract

**Background:**

Posterolateral corner (PLC) reconstruction of the knee involves precise drilling of a single tunnel from anterolateral to posteromedial in the fibular head (FH) to ensure adequate graft fixation. Misplacement of the tunnel in a too lateral or too superior trajectory can lead to cortical breach and inadequate graft fixation.

**Purpose:**

To (1) determine the mechanical consequence of a lateralized FH tunnel in PLC reconstruction and (2) compare the mechanical strength of 3 salvage fixation techniques for a lateralized FH tunnel.

**Study Design:**

Controlled laboratory study.

**Methods:**

Sawbones models of a uniform density were utilized. FH tunnels (7-mm diameter) were drilled from anterolateral to posteromedial in positive controls (lateral cortex thickness, 7.6 ± 0.7 mm) to represent an improperly placed FH tunnel at risk of lateral cortical breach. For negative controls and salvage experimental groups, FH tunnels were drilled from anterolateral to posterolateral (lateral cortex thickness, 2.7 ± 0.9 mm). Three salvage fixation techniques were compared: suture anchor fixation, tunnel redrilling, and nitinol staple fixation. Samples (n = 5 per group) underwent uniaxial tension testing, and the ultimate tensile strength (UTS) and mode of failure were recorded. Data were analyzed using the 1-sample *t* test and nonparametric 1-sample Wilcoxon signed-rank test.

**Results:**

The negative control group had a 4-fold lower mean UTS relative to the positive control group (1.49 ± 0.17 vs 6.25 ± 1.98 MPa; *P* < .01) and exhibited failure through the lateral cortex and tunnel. Nitinol staple fixation improved the mean UTS by >16 times compared with the negative control group (24.06 ± 6.49 vs 1.49 ± 0.17 MPa; *P* < .01). Suture anchors and tunnel redrilling exhibited similar UTS and mode of failure to those of negative controls.

**Conclusion:**

Reinforcement of a thinned lateral FH cortex with a single nitinol staple improved graft fixation strength in a sawbones model.

**Clinical Relevance:**

A lateralized FH tunnel can be a common intraoperative pitfall during PLC reconstruction. Salvage of a thinned lateral FH cortex with a single nitinol staple may reduce the risk of cortical breach and graft failure.

The posterolateral corner (PLC) of the knee is a complex of structures that include the lateral collateral ligament (LCL), popliteofibular ligament (PFL), posterolateral capsule, and popliteal tendon.^15,16^ PLC injuries occur from high-energy trauma involving hyperextension and varus or external rotation force, and injury to these structures can cause varus and rotational instability of the knee.^9^ Injuries to the PLC frequently occur in combination with other ligamentous knee injuries, making accurate diagnosis and subsequent management challenging.^8^

Several surgical techniques for PLC reconstruction have been described.^1,11^ In the majority of techniques, a tendon graft is traversed through a tunnel in the fibular head (FH) to reproduce the LCL and/or PFL limbs. This tunnel is created in an anterolateral-to-posteromedial fashion in the FH in order to maximize tunnel length and construct stability, as well as to reproduce the anatomic insertions of the PLC anatomy. However, because of the relatively small diameter of the FH, FH fracture can occur after PLC reconstruction.^11^ A common intraoperative pitfall is misplacement of the tunnel, either too lateral (not oblique enough) or too superior, within the FH. This may result in a thinned lateral cortex and subsequent cortical breach or fracture, resulting in graft fixation failure.

Intraoperative salvage techniques in the setting of a thinned lateral cortex or tunnel breach have not been well described. Given the potential catastrophic impact of FH tunnel breach on graft fixation and outcomes of PLC reconstruction, this study aimed to describe and evaluate the mechanical strength of 3 surgical salvage techniques for lateral misplacement of the FH tunnel in a sawbones model: suture anchor fixation, tunnel redrill, and nitinol staple fixation. It was hypothesized that laterization of the FH tunnel would negatively affect the mechanical properties of the lateral cortex and that the mechanical properties of a thinned lateral cortex could be improved by applying a salvage technique.

## Methods

### Femoral Head Tunnel Drilling and Reinforcement

**Figure 1.**
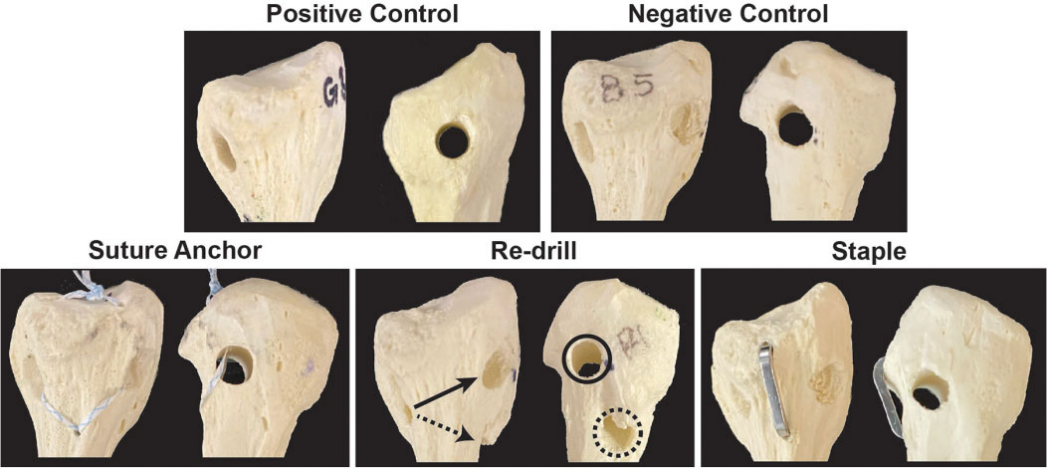
Positive and negative control fibular head tunnels, as well as suture anchor, redrill (solid arrow/circle points to original tunnel; dashed arrow/circle points to newly created tunnel), and staple salvage fixation methods.

Fibular models (sawbones) of identical density and surface topography were detached from the tibia and used for the PLC reconstruction model. Our model is in accordance with demonstrated uses for sawbones in comparative investigations that are not subjected to bending or torque for this proof-of-concept study.^3,6^ Two groups of fibular models were created. Positive control FH tunnels 7 mm in diameter^7^ were drilled starting at the sulcus where the distal LCL inserts in an anterolateral-to-posteromedial direction at the level of the champagne glass drop-off of the FH. This produced an average lateral cortical thickness of 7.6 ± 0.7 mm to represent a properly drilled FH tunnel (Figure 1). Negative control FH tunnels were drilled starting at the same starting point but at a less oblique, anterolateral-to-posterolateral direction. This produced an average remaining lateral cortical thickness of 2.7 ± 0.9 mm to represent an improperly placed FH tunnel at risk for lateral cortical breach (Figure 1). The thickness of the remaining lateral cortex was measured by calipers between the reamed tunnel and the outer surface of the fibular cortex.

Lateralized FH tunnel samples (n = 5 per group) were also used to test 3 salvage fixation methods: reinforcing the suture anchor, redrilling the tunnel in a different trajectory, or applying a nitinol staple (Figure 1). In the suture anchor group, a single-loaded 1.4-mm all-suture anchor with tape suture (Iconix with XBraid TT; Stryker) was placed distal to the lateralized tunnel in the fibular neck. The 2 tails of the tape suture were then passed through the tunnel and proximal FH in a mattress fashion and tied. In the redrill group, the tunnel was redrilled from the same anterior entry point of the FH following an anterolateral-to-posteromedial and distal trajectory, yielding a new lateral cortical thickness of 4.9 ± 0.8 mm (Figure 1). In the staple group, a single 15 × 15–mm compressive nitinol staple (DynaNite Nitinol staple; Arthrex) was placed in the lateral fibular neck and head, with the tines of the staple surrounding the top and bottom of the lateralized tunnel (Figure 1).

A single braided nylon rope 7 mm in diameter was passed through each FH tunnel to replicate a tendon graft in PLC reconstruction. Use of thawed tibialis allografts (Joint Restoration Foundation) was initially attempted for this study; however, the majority of tibialis grafts plastically elongated at low tension levels during uniaxial mechanical testing, even with preconditioning of grafts. Nylon braided rope allowed for uniform thickness and tension to be applied without deformation. Thus, this study switched to the use of uniform rope instead of tendon allografts to allow for reproducible testing of the salvage techniques and fair comparison among groups.

### Mechanical Testing

**Figure 2.**
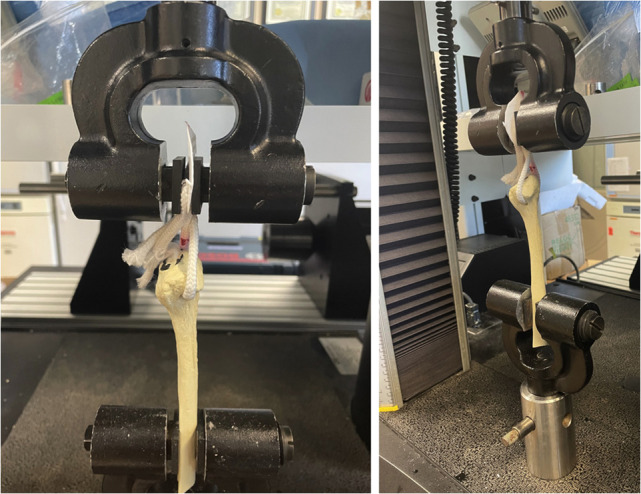
Mechanical testing setup of fibula and rope loaded in mechanical testing machine (5980 Series; Instron). Uniaxial tension was applied in line with the long axis of the fibula.

After passing the rope through the FH tunnel, the 2 ends were fixed to a plexiglass mount to ensure consistent rope lengths and positions in the proper orientation of simulated native popliteal and LCL femoral origins (Figure 2). The posterior limb of the rope reproduced the popliteal limb, and the anterior limb of the rope reproduced the LCL limb, as performed with Arciero-type PLC reconstruction.^1^ Interference screws in the FH tunnel were not placed in order to avoid further weakening and perforation of the lateralized tunnel. The mount was immobilized in the grips of a mechanical testing machine (5980 Series; Instron). The base of the fibula was immobilized such that the long axis of the fibula was oriented vertically, and the force vector applied was in line with the long axis of the fibula. Samples underwent uniaxial tension testing at 1 mm/s until failure of the sawbones, rope, or fixation apparatus. The force at failure was normalized to the cross-sectional area at the interface between the 2 broken sawbone fragments to yield the ultimate tensile strength (UTS).

### Statistical Analysis

First, a ROUT test in Prism 9 (GraphPad Software) was used to remove outliers from all groups. Then, a Shapiro-Wilk test was used to test all data for normality. Because a sawbones model of PLC reconstruction was used, all data were normalized to controls to examine the relative relationships between the groups. In the first phase, negative control UTS was normalized to positive control UTS. A 1-sample *t* test was used to compare the normalized negative control data to the negative control value. In the second phase, suture anchor, redrill, and staple UTS data were normalized to the negative control. One-sample *t* tests were used to compare the normalized suture anchor and staple UTS data with the negative control group. A nonparametric 1-sample Wilcoxon signed-rank test was used to compare the normalized redrill UTS data with the negative control. For both phases, a sample size of 5 was used.

A post hoc power analysis (α = .05) for the first phase demonstrated that the achieved power was >0.999. For the second phase, a post hoc power analysis (α = .05) demonstrated that the achieved power was 0.997 for the comparison of the suture anchor with negative control groups, as well as the comparison of the staple with negative control groups. The achieved power for the comparison of the redrill with negative control group was 0.052, with a sample size of 26 needed to achieve a power of 0.80. Because of the small difference in UTS between the redrill and negative control groups, further testing was not performed.

## Results

**Figure 3.**
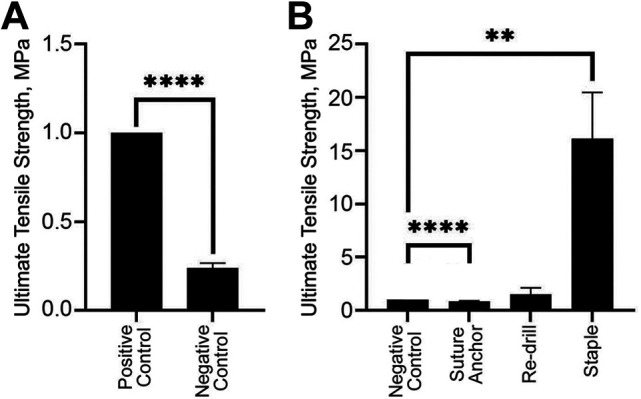
(A) Ultimate tensile strength of the negative control normalized and compared with the positive control fibular head tunnels. (B) Ultimate tensile strength of the suture anchor, redrill, and staple salvage fixation methods, normalized and compared with the negative control. Statistically significant differences: ****P < .0001, **P < .01.

**Figure 4.**
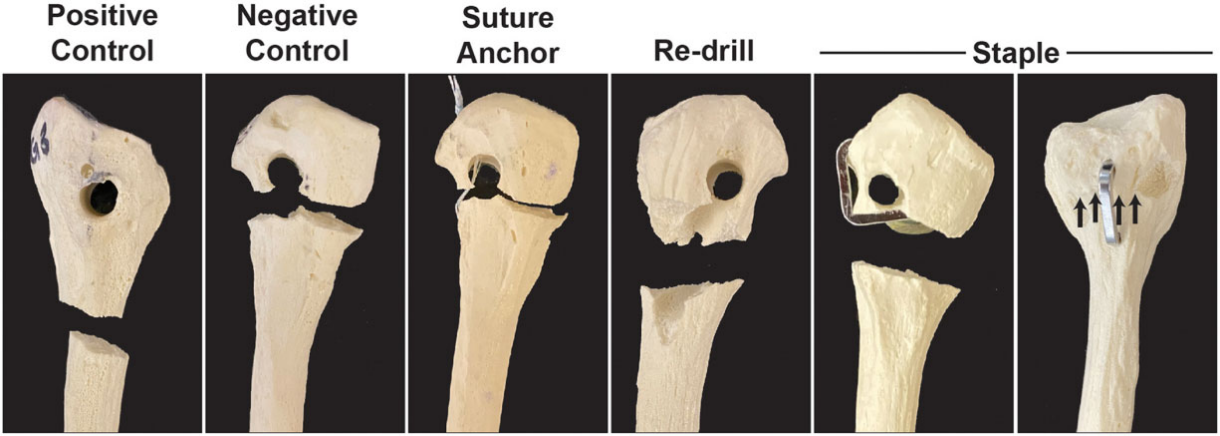
Modes of failure within the control groups and salvage fixation techniques. Arrows point to the fracture line in the lateral cortex within the staple group.

All nonnormalized tensile strength data are available in Table 1. In the first phase, the mechanical effect of a lateralized FH tunnel was determined. A lateralized FH tunnel (the negative control) significantly reduced the UTS compared with a properly drilled tunnel (the positive control) (*P* < .01) (Figure 3). Failure of the positive control group occurred at the FH, whereas failure in the negative control group occurred via fracture through the thinned lateral cortex (Figure 4).

**Table 1:** Ultimate Tensile Strength of Fibular Head Tunnel Controls and Lateral Cortex Fixation Methods*^a^*

	Ultimate Tensile Strength, MPa
Positive control	6.25 ± 1.98
Negative control	1.49 ± 0.17
Suture anchor	1.28 ± 0.07
Redrill	2.25 ± 0.94
Staple	24.06 ± 6.49

*^a^* Data are presented as mean ± SD.

In the second phase, the ability to mechanically salvage the effect of a thinned lateralized tunnel with lateral cortex fixation methods was assessed. The suture anchor fixation group had a significantly lower UTS (by 14%) compared with the negative control (*P* < .01), although this difference was small (Figure 3). The use of a single nitinol staple significantly increased the UTS compared with the negative control (*P* < .01). Failure in all the samples for the suture anchor group occurred through the thinned lateral cortex. Failure in all samples of the redrill group occurred through the posterior exit of the redrilled tunnel. Failure in the staple group occurred either through the bottom tine of the staple in the fibular neck (2 samples) or via nondisplaced fracture through the lateral cortex (3 samples).

## Discussion

This study showed that improper lateral drilling of the FH tunnel during PLC reconstruction of the knee can cause significant mechanical weakening of the lateral cortex and lead to fracture and subsequent graft fixation failure. In a sawbones model, FH tunnel lateralization, with reduction of the lateral cortex from 7.6 ± 0.7 to 2.7 ± 0.9 mm (negative control), resulted in a decrease in the UTS of the fibular tunnel with uniaxial mechanical testing and exhibited failure through the lateral cortex and tunnel. Specifically, the negative control group had a mean UTS (1.49 ± 0.17 MPa) that was 4 times lower than that of a properly drilled FH tunnel (positive control group) (6.25 ± 1.98 MPa) (*P* < .01). This study also showed that reinforcement of the lateral cortex with a single nitinol staple placed in the fibular neck and head improved the UTS to levels even above properly drilled (anterolateral-to-posteromedial) tunnels. Salvage of a lateralized FH tunnel with a single nitinol significantly increased the mean UTS by >16 times compared with the negative control group (24.06 ± 6.49 and 1.49 ± 0.17 MPa, respectively; *P* < .01). Therefore, nitinol staple fixation may be capable of salvaging a lateralized FH tunnel in PLC reconstruction.

The accuracy of the anterolateral-to-posteromedial trajectory of the FH tunnel to obtain a centralized, well-bordered tunnel is crucial to graft fixation and successful outcomes of PLC reconstruction. However, familiarity with this procedure within the surgical community is highly variable, with <14% of orthopaedic sports medicine surgeons managing >10 PLC injuries per year^5^ and even fewer surgeons performing PLC reconstruction routinely. Correct drilling of the FH tunnel during PLC reconstruction requires a high level of precision, a good understanding of the PLC and FH anatomy, careful neurolysis of the peroneal nerve, and clearance of all soft tissues along the posterior aspect of the FH to the level of the tibiofibular joint.^1,7^ In our experience, a common pitfall when drilling the FH tunnel is to place the guide pin in a trajectory that is too lateral, creating a thin lateral cortex after reaming. When the graft is placed through the lateral tunnel and tensioned, failure through this thinned lateral cortex can occur. Methods to treat this pitfall have rarely been described, and this study found that insertion of a single, low-profile, compressive nitinol staple may be a good salvage technique to reinforce and prevent breakthrough of a thin lateral cortex of the FH.^2^ Nitinol staples are an increasingly utilized product in orthopaedic procedures because the pseudoelastic properties allow for sustained compression.^10,13,17^ For example, nitinol staples have been shown to be superior to traditional fixation methods in scaphoid fractures and have great efficacy in midfoot and hindfoot arthrodesis.^4,12,14^ Furthermore, these nitinol staples are low profile and can be seated flush to the cortical surface, thereby limiting the risk of contact and irritation to the adjacent peroneal nerve. We recommend being cautious when choosing the size of the staple and ensuring the tines do not violate the proximal tibiofibular joint.

In contrast, use of a suture anchor to reinforce the lateral cortex significantly decreased the UTS of the FH by 14%. It is possible that drilling additional holes, even small holes, adjacent to the tunnel in the FH may lead to stress risers that can increase the risk of an FH fracture. In this study, a 1.4-mm all-suture anchor was used. Suture anchors in the FH or neck may be utilized to repair avulsed structures such as the fibular end of the LCL, PFL, or biceps femoris. As a result, if an FH tunnel is created, surgeons may consider placing suture anchors with enough of a bone bridge away from the tunnel to avoid weakening it or avoiding use of suture anchors altogether by suturing the avulsed structures to the PLC graft.

### Limitations

The use of a sawbones model presents several disadvantages, including the lack of a material distinction between cortical and cancellous bone, material properties that do not exactly replicate those of human bone, and the lack of reproducible tibiofemoral and proximal tibiofibular joint kinematics. However, a sawbones model provided uniform properties among samples, and the results were presented as relative values, not as absolute values, to examine the relationships between groups. To further analyze fixation methods and quantify biomechanical improvements in absolute terms, cadaveric testing may be warranted. A cadaveric model also provides the advantage of containing an intact proximal tibiofibular joint, allowing for the tensile moduli and UTS of the fixation methods to be determined. Additionally, rope was utilized instead of tendon grafts, which does not replicate normal graft mechanics. Last, continuous uniaxial tensile testing was performed, rather than a cyclical varus or rotational loading protocol that would better simulate knee loading conditions after PLC reconstruction. However, the typical mode of FH fracture through the thinned lateral cortex is caused by tension imparted by the PLC graft, and therefore, uniaxial tensile testing was utilized in this study for proof of concept.

## Conclusion

Reinforcement of a thinned lateral FH cortex with a single nitinol staple improved graft fixation strength in a sawbones model. Salvage of a thinned lateral FH cortex with a single nitinol staple may reduce the risk of cortical breach and graft failure during PLC reconstruction.
